# An analysis of the success of fiscal adjustment in reducing public debt: Evidence from Pakistan

**DOI:** 10.1371/journal.pone.0269536

**Published:** 2022-06-06

**Authors:** Ibrar Hussain, Jawad Hussain, Hazrat Bilal

**Affiliations:** 1 Department of Economics, University of Malakand, Chakdara, Dir (Lower), Khyber Pakhtunkhwa, Pakistan; 2 Department of Commerce and Management Sciences, University of Malakand, Chakdara, Dir (Lower), Khyber Pakhtunkhwa, Pakistan; 3 Center for Management and Commerce, University of Swat, Swat, Khyber Pakhtunkhwa, Pakistan; Universiti Malaysia Sabah, MALAYSIA

## Abstract

This study seeks answers to questions such as: what is fiscal adjustment? Which fiscal strategy will result in a reduction in public debt liabilities? In the pursuit of answers to these questions, the study has defined two objectives. Firstly, the fiscal adjustment episodes must be identified in order to detach the discretionary fiscal stance; secondly, the success of these adjustment episodes in reducing public debt liabilities must be assessed. As a result, attempts will be made to undertake analyses that would simplify the issues underlying Pakistan’s practical policy options. A total of eleven adjustment episodes have been observed in a sample, ranging from 1976 to 2017, following Alesina and Ardagna’s definition. The descriptive analysis reveals that five episodes succeeded in reducing the public debt, while six episodes failed to reduce the ratio. Out of the five successful episodes, four are found to be spending-based and one is tax-based. To quantify the success of fiscal adjustment, the empirical model has been calibrated on Leibrecht and Scharler’s model and estimation is done via both the Ordinary Least Squares (OLS) and Robust Least Squares (RLS) methods. The RLS method produces better outcomes than the OLS method. Under RLS, all variables are significant except GDP growth, whereas in the OLS model, the election year and regime shift, together with GDP growth, are statistically insignificant. The fiscal adjustment’s composition reveals that spending-based consolidation boosts the chances of the fiscal adjustment’s success. Fiscal authorities should, therefore, adopt spending-based austerity measures to ensure the sustainability of public finances and prevent the negative macroeconomic consequences of unsustainable public debt.

## 1. Introduction

Pakistan, being a developing country, has been confronted with the enigma of unsustainable public finance since its inception in 1947. At the beginning of the twenty-first century, the focus has been shifted to fiscal consolidation measures. In this regard, the government implemented the famous fiscal responsibility and debt limitation act in 2005 in order to bring sustainability to public finance. Since then, several efforts to reduce the budget deficit to a tolerable level have been implemented. Such efforts include both revenue and spending-based consolidation measures. But, unfortunately, the issue was not resolved and the fiscal position became more aggravated over time and with respect to regime changes in the country. Some random elements like the flood of 2010, internal insurgency, and acute energy crises also put pressure on the public exchequer and added to the fiscal deficit. However, prudent expenditure management in the face of rising revenue receipts has successfully reduced fiscal deficit from 8.2 percent of gross domestic product (GDP) in fiscal year 2013 to 4.6 percent in 2016. During the same time period, total expenditures fell from 21.5 percent of GDP to 19.9 percent, while total revenue increased from 13.3 percent to 15.3 percent [1].

Such austerity measures were initiated by the ruling Pakistan Muslim League (N) in 2013. Due to the election cycle, the fiscal deficit increased from 4.6 percent in 2016 to 6.5 percent in 2018. Because of this, the primary fiscal deficit also deteriorated from 1.6 percent to 2.2 percent of GDP during the same period. Official statistics indicate that fiscal strategy in the country has largely been imprudent for most of the time, with capital spending standing at 4.7 percent as compared to current spending at 16.5 percent of GDP. The revenue-raising efforts have also increased total revenue, which has remained flat over the last five years at 14.9 percent on average. Further investigation reveals that tax revenue accounts for 11.8 percent of GDP on average, while non-tax revenue accounts for 3.1 percent [2]. In summary, government statistics show certain serious imbalances in fiscal discipline, which may be attributed to the country’s recent macroeconomic imbalances.

In Pakistan, the current public debt management aims to ensure the financing needs and repayment obligations of the government at the lowest cost. The strategy includes primary fiscal surpluses, low and stable inflation, higher economic growth, and a market-based exchange rate regime. Besides this, the government also focuses on domestic resource mobilization and efficient spending strategies to reduce public debt liabilities. Such actions indicate a clear insight into debt sustainability in Pakistan. The debt burden of a country can be expressed in two ways: the stock ratio, represented by the debt to GDP ratio, or the flow ratio, represented by the debt to revenue ratio. Although the stock ratio has been widely recognized across the globe to measure debt sustainability, it is the flow ratio that accurately measures the repayment capacity of a nation. In Pakistan, official statistics indicate that in 2018 the economy was confronted with the problem of rising public debt, a huge circular debt with high all-round current and fiscal deficits at the pace of depleting foreign exchange reserves. In this regard, the persistent fiscal deficit has become the main source of unsustainable public debt and the resulting macroeconomic imbalances in the country. As a result, the government agreed to a $6.0 billion 39-month Extended Fund Facility with the International Monetary Fund [3].

Pakistan’s budgetary condition has deteriorated over the past three decades, and one factor contributing to this deterioration is the provision of subsidies to the power sector. Low level of national savings, growing government debt, a fluctuating currency rate, a prolonged trade deficit, and inflation are now being associated with a large fiscal deficit due to an unfavorable financing mix. Throughout the 1970s, the fiscal deficit (on average) remained at 7.1 percent of GDP, before falling to 7.0 percent of GDP in the 1980s. This downward trend continued, with the ratio falling to 6.8 percent in the 1990s and even lower to 4.8 percent in the 2000s. In contrast, the consolidated budget deficit was 4.7 percent in fiscal year 2012–13, down from 8.5 percent the previous year [4]. Rising fiscal deficits as a result of crippling energy crises are thus seen as key roadblocks to the country’s economic recovery and long-term progress [5].

High fiscal and current account deficits have put an unprecedented strain on the economy in fiscal year 2018–19 on both the domestic and international fronts. Faced with depleting foreign currency reserves, public debt has escalated to the point that debt payment consumes a sizable portion of the government budget. Recent macro imbalances have been linked to economic and structural constraints that have been largely neglected for decades due to poor policy responses [2]. According to official statistics, the economy grew at a 4.7 percent annual rate over the previous five years, falling short of the 5.4 percent predicted. Unplanned and wasteful expenditures, along with poor income growth, slow exports, and surging imports, has resulted in the country’s twin-deficit conundrum. The economy expanded at a paltry 3.29 percent in fiscal year 2018–19, compared to a modest target of 6.2 percent. Aside from that, total investment was 15.4% of GDP, while total saving was 10.7%, resulting in a 4.7 percent difference.

Furthermore, according to official statistics [2], the average fiscal deficit throughout the Pakistan Muslim League (N)’s term was 5.6 percent. The average total revenue was 14.9 percent of GDP, whereas the average total spending was 20.5 percent. In 2018–19, inflation averaged 4.8 percent, but jumped to 7 percent in the first year of the newly elected government. Aside from that, the current account deficit in 2017–18 was $19.9 billion, or about 6.3 percent of GDP. In the current period, Pakistan’s revenue structure is shifting substantially from indirect to direct taxation. Inconsistent macroeconomic policies in the face of political instability, along with random incidences such as the flood of 2010, and internal insurgency, have resulted in limited fiscal space. Therefore, all such reinforcing factors of the fiscal deficit in the country necessitate further research into the issue. Crucially, the aim is approached in two ways. To ascertain the real fiscal stance of the government, it is first necessary to identify episodes of fiscal adjustment. Second, it is important to analyze the success of these adjustment episodes in terms of decreasing public debt liabilities. Accordingly, efforts will be directed toward analyses that must be simplified in order to better understand the issues underlying Pakistan’s real policy options.

## 2. Review of literature

This section focuses on the theoretical underpinnings of the success of fiscal policy. Theoretical and empirical components of fiscal policy, as well as their macroeconomic effects, might benefit from such discussion. This section also includes a brief discussion of the built-in opportunities and challenges in the fiscal policy transmission mechanism.

Growing public debt as a result of policy blunders and electoral distortions has been claimed to necessitate significant budget deficit reduction across the country. In the literature, such a significant decrease in the budget deficit is referred to as "fiscal austerity" [6]. Aside from this, many countries in Europe and the OECD have implemented austerity initiatives in 2010. This has sparked a relatively heated debate, with one side supporting the policies and the other declaring them to be self-defeating measures. Since then, a substantial body of literature has developed around Giavazzi and Pagano’s significant writings [7]. The resulting literature contradicted the predictions of neo-classical and Keynesian models about the impact of fiscal austerity on the macro economy. In contrast to the typical Keynesian expectation of a recession caused by fiscal contraction, this line of study revealed that such fiscal contraction was expansionary, leading to the coining of the phrase "expansionary fiscal contraction." According to Giavazzi and Pagano’s findings, in a scenario where the cost of public debt is high, a drop in the cost of public debt through fiscal consolidation leads to an expansionary fiscal contraction. Because of expectations about a future tax decrease, fiscal consolidation helps to boost private consumption by encouraging people to spend more money. The idea had a significant impact on policymakers all over Europe when it came to developing budgetary adjustment methods [8].

Following in the footsteps of Giavazzi and Pagano, other studies have been conducted, including those by Alesina and Perotti [9, 10], Ardagna [11], Alesina et al. [12], and Hussain et al. [13]. As a result, a group of researchers has come to an agreement on the best way to design the budgetary changes. According to Alesina and Perotti [10], the structure of the fiscal adjustment affects the possibility of permanent budget consolidation and macroeconomic impacts. The study further identifies the two types of fiscal adjustment and differentiates type-I from type-II fiscal adjustment. Cuts in spending, particularly transfers, government employment and wages, and social security, are the key components of Type-I adjustment. The type II adjustment, on the other hand, is connected to a broad-based tax increase, including increases in family taxes and social security payments. In the influential study by Alesina and Ardagna [14], two critical questions have been pursued regarding fiscal adjustments, the first related to the effective mix of spending cuts and tax increases in order to achieve sustainability in the debt-to-GDP ratio, while the second one is focused on the employment and output losses of fiscal adjustment. According to Cottarelli and Jaramillo [15], governments under limited market pressure should make modest budgetary adjustments to underpin a coherent strategy for long-term public finance and economic growth.

According to the literature that has been studied so far, there are variations of opinion about the nature of fiscal adjustment and its macroeconomic repercussions. However, a group of important economists has reached a consensus on the function of fiscal adjustments in setting a country’s public finances on a sustainable path and limiting negative consequences. It’s important to understand whether fiscal policy changes occur during a recession or an expansion in order to make sound decisions. As a result, many governments are implementing expenditure-based adjustments during booms and tax-based consolidations during recessions. It has also been found that spending-based adjustment is far more effective than tax-based adjustment [7, 14, 16]. It seems more subtle, but Alesina et al. [16] contend that economic cycles may have a hand in the decision between the two types of adjustment.

There is a scarcity of empirical research on fiscal consolidation in developing countries such as Pakistan, but there are an expanding number of studies on fiscal consolidation in industrialized countries. Even if a country’s unique circumstances define the right composition of fiscal adjustment, policymakers should examine the long-term impact of the chosen fiscal policies on growth and equity [17]. According to Krugman [18], public debt has a detrimental influence on economic growth because it increases uncertainty about future taxation and weakens the country’s ability to withstand macroeconomic shocks. Evidence suggests that the level of public debt is likely to have a negative impact on growth and, in this regard, the country’s investor base, besides the degree of development, plays an important role [17]. Based on the country’s level of development and investor base, evidence shows that the amount of public debt is likely to have a negative influence on growth [17]. In a nutshell, this body of literature highlights the important macroeconomic effects of fiscal consolidation in many economies, regardless of their degree of development. According to Alesina and Perotti [10], the composition of the fiscal adjustment matters for its macroeconomic effects for three reasons. Aside from the labor market effect, the study investigates the effects of expectation and political legitimacy on the repercussions of fiscal consolidation. As a result, policymakers should consider the long-term viability of consolidation policies as well as their implications for growth and equity [17]. Many analytical methodologies with solid theoretical underpinnings may be utilized to examine the impact of fiscal changes on a nation’s public finances, which should not be disregarded in the present endeavor, especially in a resource-constrained country like Pakistan.

## 3. Materials and methods

Identifying FA episodes and isolating the discretionary fiscal stance has been a subject of debate among scholars. An episode of FA, according to Alesina and Perotti [9], is defined as an annual improvement in CAPB of at least 1.5 percentage points, with success measured by a decline in the debt-to-GDP ratio of up to 4.5 percentage points over the next three years. Alesina and Ardagna [19] have proposed similar definitions. In a number of other studies [11, 20, 21], FA was defined as a change of at least 2% in one year or at least 1% in two consecutive years. Ahrend, Catte, and Price [22], as well as Guichard, Kennedy, Wurzel, and André [23], agree that FA execution will begin if CAPB improves by at least 1 percent of potential GDP in one year. These authors also contend that FA will commence if the ratio improves by at least 0.5 percent in the first two successive years and will continue as long as CAPB rises by 0.3 percent. However, if the increase falls below 0.2 percent of GDP, it will be phased out.

The current analysis, as indicated below, aligns with Alesina and Ardagna’s [14] concepts of fiscal adjustment and its success in reducing public debt:

**Definition-1:** A fiscal adjustment episode is defined as a two-year period during which the CAPB-to-GDP ratio rises each year by at least two percentage points.
**Definition-2:** A fiscal adjustment episode is considered successful if it reduces the debt-to-GDP ratio; more specifically, if this ratio–two years after the end of fiscal adjustment–remains below the ratio in the fiscal adjustment’s final year.

### 3.1 Methodology for the success of the fiscal adjustment

When attempting to quantify the success of adjustment, the current study’s approach has been calibrated on the Leibrecht and Scharler [24] model, which is explained as follows:

Redt=λ0+λ1Xt+λ2Comt+εt (1)

The term “Red_t_” refers to the actual percentage point decrease in the debt-to-GDP ratio, not a threshold at which an adjustment is declared effective. This investigation would use OLS, subject to the underlying assumptions and Robust Least Square, analogous to Leibrecht and Scharler’s [24] work. The letter “X_t_” is a vector of control variables and would include the type of government, average fiscal deficit three years prior to adjustment, GDP growth, and exchange rate regimes. The term “Com_t_” is the variable that captures the composition of adjustment and is constructed by the sum of the change in Hodrick-Prescott (HP) filtered primary public spending as percent of the GDP and the change in HP filtered total revenue as percent of the GDP. Many influential studies [11, 14, 24] have used this variable in their analyses.

## 4. Results and discussion

This section sheds light on the identification of fiscal adjustment episodes in Pakistan, their success in reducing public debt liabilities, and also compares selected macroeconomic indicators during successful and unsuccessful episodes. Aside from these, the macroeconomic effects of the composition of fiscal adjustment and econometric evidence have been thoroughly discussed.

### 4.1 Identifying the fiscal adjustment episodes

This study adopts and relies on the definitions thoroughly discussed by Alesina and Ardagna [14] in their study. Over the entire sample period from 1976 to 2017, eleven episodes have been identified. An episode of fiscal adjustment consists of two consecutive years in which the cyclically adjusted primary balance (CAPB) improves each year. Furthermore, the sum of this ratio should be equal to two points of the CAPB as a percentage of GDP. The majority of existing FA identification research, on the other hand, has been done for OECD or European nations. As a result, the average government size in these countries, as measured by public spending, remained at 40% of GDP. In Pakistan, however, the average level of government spending over the entire sample period is 22.84 percent, and when cyclically adjusted, it is only 17.69 percent [13]. Following Hussain et al. [13], each year’s change in the CAPB was scaled by the cyclically adjusted primary spending for that year. The justification for such scaling is based on the impact that a larger government has on the country’s current fiscal policy: the larger the government, the simpler it is to change fiscal policy.

The identification of FA episodes based on Definition-1 is summarized in Table 1. Based on Definition-2, this table distinguishes between successful and unsuccessful episodes. A further contrast between the two types of adjustments is shown in the same table. According to Devries et al. [25], an episode is defined as expenditure-based if the absolute drop in cyclically adjusted primary spending surpasses the increase in cyclically adjusted revenue. CAPS denotes cyclically adjusted primary expenditure in the study, whereas CAR represents cyclically adjusted revenue. Successful episodes reduce the debt-to-GDP ratio significantly, but unsuccessful episodes do not. Five episodes are found to have succeeded, while six episodes are deemed to have failed, according to Definition-2. Four of the five successful episodes are based on spending, while one is based on taxes. According to observation, unsuccessful episodes are half spent and half taxed.

**Table 1:** Distinction between successful and un-successful episodes.

Nature of Episode	Spending-Based Fiscal Adjustment	Tax-Based Fiscal Adjustment
**Successful Episode**	1991–1992	1977–1978
	1993–1994	-
	1997–1998	-
	1999–2000	-
**Un-Successful Episode**	1981–1982	1979–1980
	1989–1990	2013–2014
	1995–1996	2015–2016

### 4.2 An account of the success of fiscal adjustment

Five of the eleven episodes listed in Table 2 succeeded in lowering the public debt ratio, while the other six did not. The first successful episode (1977–78) reduced the public debt as a percentage of GDP from 63.485 to 52.121, making it the most successful of all episodes, while the second most successful episode (1999–2000) reduced the ratio from 82.904 percent to 75.764 percent, making it the second most successful of all episodes. During these periods, the military assumed control of the government and implemented budgetary austerity measures. In the first episode, General Zia ul Haq, the then-military chief of staff, assumed command of the government; in the second, General Musharraf imposed martial law. The other three episodes took place during democratic administrations.

**Table 2:** Reduction in debt-to-GDP ratio (Definition-2).

Un-Successful Fiscal Adjustment				Successful Fiscal Adjustment			
Episodes	FA_LY_ (1)	After (2)	Change (2)-(1)	Episodes	FA_LY_ (1)	After (2)	Change (2)-(1)
1979–80	62.253	82.365	20.112	1977–78	63.485	52.121	-11.364
1981–82	58.305	65.444	7.139	1991–92	80.401	80.228	-0.173
1989–90	83.066	85.144	2.078	1993–94	85.837	82.156	-3.681
1995–96	80.371	100.259	19.888	1997–98	89.332	87.509	-1.823
2013–14	62.952	67.100	4.148	1999–00	82.904	75.764	-7.140
2015–16	67.700	74.200	6.500	--	--	--	--
Average	69.107 (4.184)	79.085 (5.321)	9.977 (3.253)*	Average	80.392 (4.482)	75.556 (6.154)	-4.836 (2.001)**

Note: Standard errors are reported in parentheses. While * and ** show the difference is significant at 5% and 10% probability levels, respectively, FA^LY^ indicates the last year of fiscal adjustment, whereas "After" represents two years after the fiscal adjustment.

In research undertaken in Chile, Schmidt-Hebbel [26] contends that, in addition to strong institutions, it is the military government that can implement fiscal reforms in their true spirit. The average debt ratio reduction during successful episodes is 4.836 percentage points, compared to an average increase of 9.978 percentage points during unsuccessful episodes, and both reductions are significant at the 10 percent and 5 percent probability levels, respectively. Furthermore, during the successful episodes, the average debt-to-GDP ratio in the FA’s final year was 80.392 percent, compared to 75.556 percent two years after the adjustment ended. Similarly, during unsuccessful episodes, this ratio rises from 69.107 percent to 79.085 percent.

Table 3 compares and reports on selected macroeconomic indicators during successful and unsuccessful episodes. During successful episodes, the average debt-to-GDP ratio is 81.544 percent, while during unsuccessful episodes, it is 69.196 percent. At the 5% level, this difference is statistically significant. The average debt-to-GDP ratio improves by 4.836 points when successful episodes occur, compared to a deterioration of 9.977 points during unsuccessful episodes.

**Table 3:** Macroeconomic variables during successful and un-successful episodes.

Variables	Successful	Un-successful	S. E. of Difference
Debt/GDP	81.544	69.196	4.278*
Debt/GDP_(t+2)_–Debt/GDP_(t)_	-4.836	9.977	4.865*
Growth_(t)_–Growth_(t-2)_	-0.318	0.510	1.205
GDP Growth	4.174	5.673	0.518*
Current Account Balance	-3.254	-3.854	0.538
Private Investment	8.796	8.187	0.394
Inflation (GDP-Deflator)	11.450	8.660	1.789
Unemployment Rate	4.967	3.437	0.610*
Primary Fiscal Balance	-1.807	-2.575	0.594
Overall Fiscal Balance	-7.212	-6.562	0.494
Call Money Rate	9.626	8.749	0.679
Real Effective Exchange Rate	127.474	140.537	14.206

Note: * indicates significance at 5% probability level.

Interestingly, we found this difference to be significant. The average GDP growth rate during successful episodes is 4.174 percent, while the average GDP growth rate during unsuccessful episodes is 5.673 percent. This difference is also found to be statistically significant at 5% probability. Although the difference between successful and unsuccessful episodes is statistically insignificant, they are both mildly contractionary. It has been observed that other macroeconomic indicators such as the current account balance, private investment and inflation are not affected by the success of fiscal consolidation. At this stage, fiscal balance, interest rates, and even the exchange rate do not tend to respond to the effectiveness of FA.

It has been observed that the labor market reacts strongly to adjustments, and that the average unemployment rate during successful episodes is greater than during unsuccessful episodes, with a significant difference. During successful episodes, the average primary fiscal balance is lower than during unsuccessful ones. This change, however, is insignificant. The budget deficit (overall) and interest rates (short-term) are slightly higher in successful episodes than in unsuccessful ones, although the differences are insignificant. During the successful periods, the domestic currency remained stronger than during the unsuccessful ones. This distinction is likewise determined to be insignificant.

### 4.3 Macroeconomic effects of the composition of FA

Following the definition of Devries et al. [25] regarding an adjustment being either spending-based or tax-based, this study reveals that four successful episodes are spending-based and only one is tax-based. This finding contradicts Alesina and Ardagna’s [14, 19] findings, which suggest that spending-based adjustment reduces the debt-to-GDP ratio more effectively than tax-based adjustment. Furthermore, the unsuccessful episodes are split half and half between spending and taxes. Table 4 summarizes the information about the composition of fiscal adjustment episodes based on their success.

**Table 4:** Macroeconomic effects of composition of fiscal adjustment.

Macroeconomic Indicator	Successful Episodes	Un-Successful Episodes	Standard Error
ΔPrimary Deficit	-1.682	-2.445	0.497***
ΔTotal Deficit	-6.982	-6.616	0.324
ΔTax	12.743	12.837	0.471
ΔIndirect Tax	8.398	8.762	0.231*
ΔDirect Tax	2.725	2.877	0.193
ΔSpending^¶^	23.462	22.934	0.505
ΔPrimary Spending^¶^	18.184	20.004	0.838**
ΔCurrent Spending^¶^	19.350	17.540	0.554**
ΔDev. Spending^¶^	5.549	6.093	0.306**
ΔTotal Revenue^¶^	16.179	15.896	0.312
ΔTax^¶^	12.938	12.671	0.305
ΔDirect Tax^¶^	2.755	2.863	0.158
ΔIndirect Tax^¶^	8.619	8.607	0.164**
Com-Spending	-30.107	-9.684	5.315**
Com-Current Spending	-16.811	-11.501	6.626
Com-Dev. Spending	-15.469	-4.306	3.778**
Com-Total Revenue	-9.215	8.383	5.144**
Com-Tax	-6.817	10.514	6.571***
Com-Indirect Tax	-11.487	-2.024	2.328*
Com-Direct Tax	5.861	5.844	2.912

Note: Com-Spending, Com-Current Spending, Com-Dev. Spending, Com-total Revenue, Com-Tax, Com-Indirect Tax, and Com-Direct Tax indicate the change in respective variables (cyclically adjusted) in percentage points of the change in the cyclically adjusted primary deficit.

The symbol “^¶^” indicates that the respective variable is cyclically adjusted.

The table shows that during the unsuccessful periods, the reduction in the primary fiscal deficit is greater than during the successful times. This difference is statistically significant at the 10% level. Surprisingly, there is no statistical difference in overall fiscal deficit reduction between successful and unsuccessful episodes. When comparing the average indirect taxes as a percentage of GDP between successful and unsuccessful periods, it is evident that the ratio is lower during the successful periods as compared to the unsuccessful ones, and the difference is statistically significant. While comparing the successful episodes with the unsuccessful ones, however, there is no statistically significant difference in direct taxes. Furthermore, when we compare the successful episodes with the unsuccessful ones, there is no statistical difference between the total tax collection and the cyclically adjusted total spending.

However, there is significant evidence that the successful episodes reduce the CAPS as compared to the unsuccessful ones. During successful episodes, cyclically adjusted current spending is significantly higher than during unsuccessful ones. Contrarily, cyclically adjusted development spending is lower during the successful episodes than during the unsuccessful ones, and the difference is significant. Cyclically adjusted revenue is higher during the successful episodes than during the un-successful ones, but this difference is statistically insignificant. Besides this, there is no significant difference between the cyclically adjusted taxes when we compare the successful episodes with the un-successful ones. Cyclically adjusted direct taxes remain the same when we compare the successful with the un-successful periods. Cyclically adjusted indirect taxes are significantly higher in the successful episodes than in their counterpart episodes. When the influence of FA composition is investigated, the outcome shows that spending-based consolidation plays a critical role in lowering the debt-to-GDP ratio.

This indicates one important feature of the composition of public spending in Pakistan: the government prefers to choose politically less sensitive components by reducing capital spending whenever implementing austerity measures. This is evident in political governments’ behavior, as evidenced by a significant 34 percent reduction in capital spending in fiscal years 2018–19 in order to reduce the debt-to-GDP ratio. Further observation reveals that adjustment on the current spending-side plays an important role in setting an expansionary effect but does not successfully reduce the debt-to-GDP ratio. Although not causal from descriptive analyses, the revenue-based adjustment seems to influence the success of FA, but it also carries a contractionary effect. Remarkably, this finding is in conformity with the findings of Alesina and Ardagna [19]. In a similar vein, the composition of the total and indirect taxes also increases the likelihood of FA succeeding. However, the study demonstrates that in the case of Pakistan, the structure of direct taxes has no substantial impact on the success and impact of fiscal adjustment.

### 4.4 Econometric evidence on the success of fiscal adjustment

To quantify the success of fiscal adjustment (FA), the model of Leibrecht and Scharler [24] is employed. Following Definition-2 regarding the success of FA, an episode is considered to be successful if the ratio of debt-to-GDP two years after the end of FA is lower than the ratio in the last year of adjustment. Over the sample of the study ranging from 1976 to 2017, eleven episodes have been identified, each consisting of two years. Five episodes successfully reduced the debt-to-GDP ratio, while six episodes failed. The model is estimated using the actual reduction in debt-to-GDP ratio as the dependent variable. In line with the notion of Leibrecht and Scharler [24], the variable Red in equation-1 indicates the percentage point reduction in public debt (debt-to-GDP ratio in this case) in the two years after the commencement of FA. To control for the impact of the election cycle and regime switching, two categorical variables denoted by D^EL^ and D^RG^ were introduced. Here, D^EL^ is defined as a dummy that takes the value of "1" if the preceding year of FA is an election year and "0" otherwise. Politicians often increase public spending in the last year of their tenure so as to increase their chances of being re-elected. This creates a political cycle that makes public finances unsustainable. Similarly, when a regime shift occurs, the new government usually adopts strict austerity measures in order to achieve the objective of sustainable public finance in Pakistan. The study therefore intends to control for the effect of austerity measures taken by the newly elected government via D^RG^. This would have the value of "1" if a new government takes over in the preceding year of FA and "0" otherwise.

To capture whether the composition of FA matters for its success, we adopt the approach of Devries et al. [25] and define a dummy variable (Com) with a value of "1" if the negative change in CAPS (in absolute terms) exceeds the positive change in CAR and "0" otherwise. Because it has been suggested that initial conditions might impact FA success [24, 27], the analysis used the average debt-to-GDP ratio in the two years prior to the FA, denoted by Debt, and the cumulative change in CAPB in the two years preceding the FA, indicated by Def. The introduction of these variables in the model is intended to capture the effect of fiscal distress on the likelihood of an adjustment succeeding. To ascertain the impact of the exchange rate regime on the success of FA, a categorical variable denoted by D^EX^ is taken to have a value of "1" when the exchange rate regime is flexible and "0" otherwise. The data on exchange rate regimes was obtained from Hamid and Mir’s [28] study. Furthermore, to capture the impact of the macroeconomic environment in which FA takes place, average GDP growth and average unemployment rate in the preceding two years of FA are included in the empirical model. The Dickey-Fuller GLS (also known as the ERS) test is used to assess the variables for stationarity at their levels and first differences before estimating the model. The results of the test are reported in Table 5. On the basis of the ERS test, the variables Red, Debt, Def, GDP_g_, and UE are stationary at levels with drifts.

**Table 5:** Stationarity evidence from Dickey-Fuller GLS (ERS) test.

Variables	Dickey-Fuller GLS (ERS)			
	Drift		Drift & Trend	
	Level	1^st^ Difference	Level	1^st^ Difference
Red	-2.72*	-4.20*	-2.83	-4.32*
Debt	-2.02**	-5.71*	-2.04	-6.07*
Def	-2.58**	-4.85*	-3.02***	-4.80*
GDP_g_	-1.89***	-4.64*	-2.40	-4.63*
UE	-2.42**	-4.81*	-2.57	-4.98*

Note: The probability levels of 1%, 5%, and 10% are denoted by the symbols *, **, and ***, respectively.

**Fig 1:**
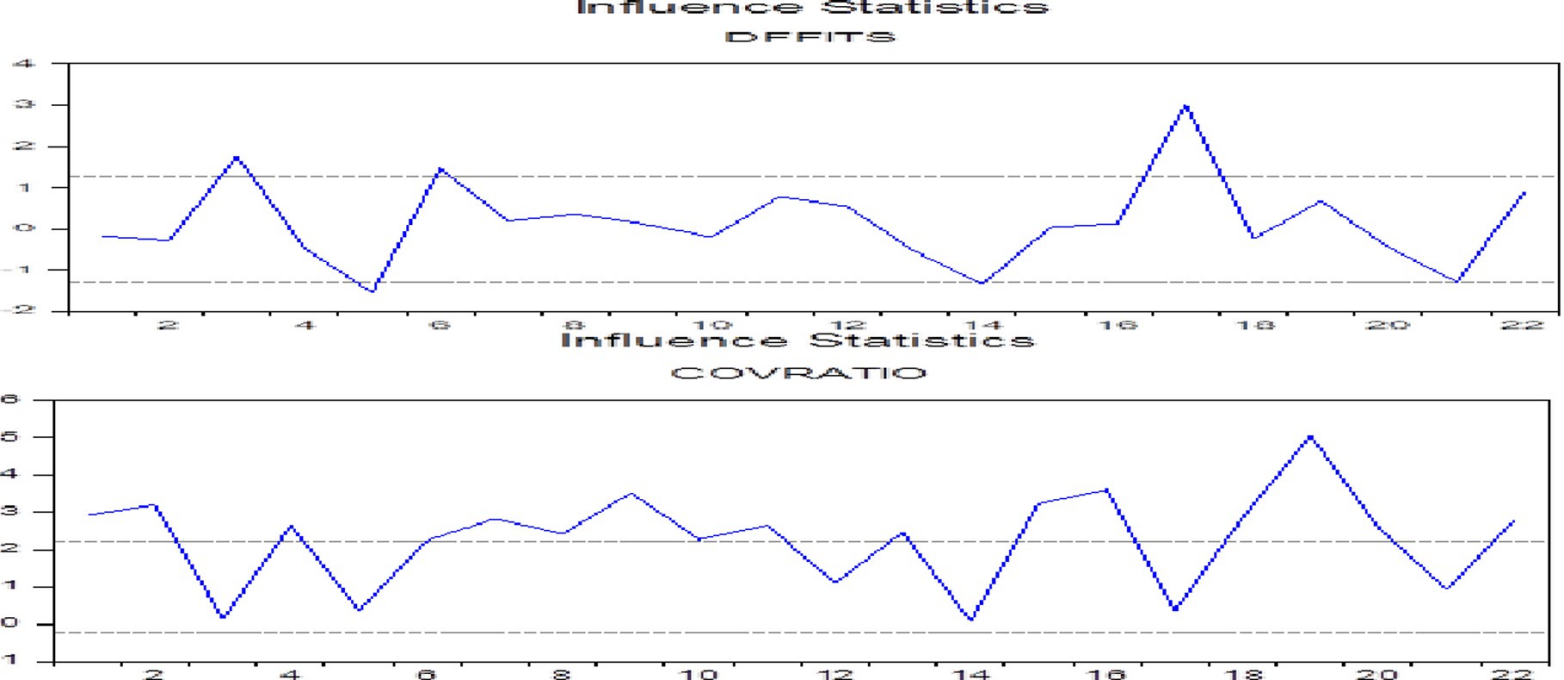
Influence statistic: Success of fiscal adjustment model. The impacts of individual data points or groups of data points on a statistical study are measured using influence statistics. The spikes in the graphs based on the two influence measurements indicate outliers in the data.

After stationarity checking, the model is estimated via OLS and reported in S1 Appendix. However, OLS estimators are not only biased but inefficient in the presence of outliers in small samples of non-Gaussian data [29]. The study therefore applies the robust Least Square (RLS) method following the influential work of Colombier [30]. The results of RLS are reported in Table 6. Researchers are often more careful about the problems of endogeneity, parameter heterogeneity, model uncertainty, and outliers [30, 31]. In contrast, outliers have gained little attention so far in empirical research [29]. Other influential authors like Yohai, Stahel, and Zamar [32], Temple [31], Zaman et al. [29], and Atkinson and Riani [33] propose the RLS method in the presence of outliers in the data. The graphs of influence statistics (reported in Fig 1) indicate the presence of outliers in the data. From both graphs, it is clear that outliers influence estimates of the OLS model.

**Table 6:** Estimates of the success of fiscal adjustment.

Variable	Robust Least Square		
	Coefficient	Standard Error	Probability
Def	33.294	5.967	0.000
Com	12.145	3.474	0.001
Debt	-91.624	15.600	0.000
GDP_g_	-4.416	3.059	0.149
UE	-11.129	3.369	0.001
D^EX^	24.087	3.418	0.000
D^EL^	-5.997	2.427	0.014
D^RG^	19.023	2.975	0.000
C	363.152	65.105	0.000

R^2^ = 0.52 Adj. R^2^ = 0.23 J.B = 2.60(0.27)

Q-Stat. = 4.76(0.09) Rn.Sq. Stat. = 77.20(0.00)

Diagnostic tests (reported in S1 Appendix) for the OLS model include the Fischer-test for overall significance, Ramsey RESET for specification, Breusch-Pagan-Godfrey (B.P.G) for heteroskedasticity, Jarque-Bera (J.B) for normality, and the Durbin-Watson (D.W) statistic for serial correlation. The probabilities of the respective test statistics are reported in parenthesis. The HAC (Newey-West) method is adopted to adjust the standard errors of the estimates. Both models pass all the diagnostic tests. A Q-statistic is used for testing serial correlation in the RLS model. Robust estimation types have been based on M-estimation in the presence of an outlier in the dependent variable with Bisquare as the objective specification function. Note here that M stands for “generalized maximum likelihood estimator.” The variables that are entered in log form are Debt, GDP_g_, and UE.

The results of RLS are better than those of OLS. All the variables are significant except GDP growth under RLS, while the influence of election year (D^EL^) and regime shift (D^RG^) along with GDP growth are statically insignificant under the OLS model. The cumulative change in CAPB in the preceding year of FA is positively related to the reduction in debt-to-GDP ratio, whereas the debt-to-GDP ratio in the preceding years of FA (representing fiscal distress) is negatively related to the actual debt reduction. These two variables were included to control for the impact of initial conditions on the success of FA. These findings do not match with the findings of Leibrecht and Scharler [24]. Their estimates show a negative effect of the deficit and a positive impact of debt in the preceding years on the actual reduction in the ratio of debt-to-GDP. However, their estimates were statistically insignificant. The positive effect of the deficit is in line with the findings of Tagkalakis [34]. In both models, the composition of FA is found to have a positive effect on actual reduction (Red). Remarkably, spending-based consolidation is found to increase the likelihood of fiscal adjustment succeeding. This finding is in conformity with the findings of Alesina and Perotti [9, 10], Von Hagen, Hallett, and Strauch [35], Alesina and Ardagna [14, 20], De Cos and Moral-Benito [36], and Yang et al. [37]. Regarding the effect of monetary and exchange rate policies on the success of FA, a strand of literature points out the direct link between exchange rate policy and the success of FA [34]. The current study finds a significant positive effect of the exchange rate regime (D^EX^) with a flexible regime that positively adds to the success of FA. This finding is not in line with the findings of Leibrecht and Scharler [24], who found a negative effect of a flexible exchange rate. However, the finding is in conformity with the findings of Lambertini and Tavares [38], Ahrend et al. [22], Tagkalakis [34], and Devries et al. [25]. In their studies, these authors argue that deprecation increases the chances of success.

The influence of the newly elected government on the success of FA is captured by introducing a dummy variable denoted by D^RG^. Under the RLS model, the associated coefficient is positive and statistically significant. This implies that FA will succeed when the newly elected government initiates austerity measures. This finding confirms the claims of Alesina, Ardagna, and Trebbi [39] regarding the positive impact of the newly elected government on the success of FA. The authors argue that newly elected governments normally opt for strict austerity fiscal measures in order to put public finances on a sustainable path. The impact of the election cycle is significant and it negatively affects the success of FA. The plausible explanation lies in the opportunistic behavior of politicians to influence election outcomes in their favor by increasing public expenditures, as indicated by Alesina and Tabellini [40] in their study. Besides this, the authors argue that the degree of polarization between the alternating governments also determines the equilibrium level of public debt. Such a situation provides an opportunity for the political party in power to use the policy of public debt accumulation to influence the choices of its successors. This is what is happening in Pakistan, where no single political party has been re-elected over the sample period. This finding of the study confirms the findings of Schuknecht [41], who conducted a study for twenty developing countries over annual data ranges from 1973 through 1992. The author arrives at the conclusion that a government’s likelihood of running an expansionary fiscal policy increases around an election and is often more prone to increasing public expenditure than lowering taxes. According to Fatas and Mihov [42], such government behavior toward expansionary fiscal policy around election years is also observed in their study; the cycles of public investment become prominent, which often lead to political business cycles. Many studies suggest institutional structures to limit the opportunistic behavior of the political party in power when it comes to fiscal policy. Another study by Hübscher and Sattler [43] came to the conclusion that vulnerable governments strategically avoid fiscal adjustment towards the end of their last year of tenure in order to minimize electoral punishment. Turning to the effect of the macroeconomic environment on the success of FA, two variables were integrated into the regression model: GDP growth and unemployment rate. The impact of the unemployment rate (UE) is significantly negatively associated with success. This indicates that the higher the unemployment rate, the less likely the episode of adjustment will succeed. This finding is in conformity with the findings of Leibrecht and Scharler [24]. Similarly, the effect of GDP growth (GDP_g_) is positive, but it is statistically insignificant under both the OLS and RLS models. This finding also supports the findings of Leibrecht and Scharler [24]. The plausible explanation lies in the fact that when the unemployment rate remains high, governments avoid consolidation measures. To unload the unemployment burden, they run an expansionary fiscal policy in order to avoid electoral punishment.

## 5. Conclusion and policy recommendations

A detailed analysis indicates that three of the five successful episodes were expansionary, two were contractionary, and only one was contractionary of the five unsuccessful occurrences. Unfortunately, the proportion of episodes that fail to expand economic activity is higher than that of successful ones, which is somewhat surprising. According to the analysis of adjustment episodes, seven of them were based on spending, whereas only four were based on taxes. Since Alesina and Ardagna’s investigations revealed that the bulk of successful and expansionary episodes are based on spending reduction, this is in accordance with their findings [14, 19]. Further investigation reveals that the majority of unsuccessful and expansionary episodes are tax-driven. Contractionary episodes were all based on spending, regardless of whether they were effective or not, and none of them was determined to be tax-based. Expansionary episodes were split equally between spending and taxation. According to the descriptive analyses, tax-based adjustments are not contractionary, despite the fact that they may fail to reduce the debt-to-GDP ratio when compared to spending-based consolidation. As a result, the twin objectives of sustainable public finance and economic growth cannot be achieved simultaneously, implying a trade-off relationship. If the goal is to get the economy moving toward economic growth, tax-based adjustments will help, while spending-based consolidation will help accomplish the goal of long-term fiscal sustainability. The average debt-to-GDP ratio for successful episodes is 81.54, whereas for unsuccessful episodes it is 68.58. This analysis also reveals an essential point: when the debt-to-GDP ratio exceeds specified upper limits, fiscal authorities resort to austerity measures.

In addition to describing fiscal adjustment, this study aimed to empirically quantify the impact of both the size and composition of fiscal adjustment on Pakistan’s debt-to-GDP ratio. The Leibrecht and Scharler [24] model is used to assess the success of fiscal adjustment (FA) in lowering the debt-to-GDP ratio. The Robust Least Squares (RLS) approach is used due to the existence of outliers in the data. The RLS model produces better outcomes than the OLS model. Under RLS, all variables are significant except GDP growth. However, under OLS, the impact of election years and regime shifts, as well as GDP growth, are statistically insignificant. The reduction in debt-to-GDP ratio in the preceding year of FA is favorably linked with the cumulative change in CAPB, but the reduction in debt-to-GDP ratio in the preceding years of FA is adversely associated with actual debt reduction. These two variables were added to account for the influence of initial conditions on FA success. In both models, the composition of the FA has a positive effect on the actual reduction of the debt-to-GDP ratio. Remarkably, spending-based consolidation tends to increase the likelihood of fiscal adjustment being successful. To ensure the sustainability of public finances and avoid the negative macroeconomic consequences of unsustainable public debt, fiscal authorities should implement spending-based austerity measures in Pakistan.

## Supporting information

S1 Data (XLSX)

S1 Appendix Estimates based on Ordinary Least Square (OLS) method. (DOCX)
