# Supplementary material for: An analysis of the success of fiscal adjustment in reducing public debt: Evidence from Pakistan
Source: PLoS One. 2022 Jun 6;17(6):e0269536. doi: 10.1371/journal.pone.0269536 (PMC9170094; doi:10.1371/journal.pone.0269536)
Supplement: S1 Appendix — (DOCX) [file pone.0269536.s002.docx]

**S1 Appendix. Estimates based on Ordinary Least Square (OLS) method**

| **Variable** | **Ordinary Least Square** | | |
| --- | --- | --- | --- |
|  | **Coefficient** | **Standard Error** | **Probability** |
| Def | 26.241 | 13.238 | 0.069 |
| Com | 14.960 | 5.242 | 0.014 |
| Debt | -88.514 | 20.189 | 0.001 |
| GDP_g_ | -2.679 | 5.579 | 0.639 |
| UE | -11.312 | 5.227 | 0.050 |
| D^EX^ | 23.220 | 6.385 | 0.003 |
| D^EL^ | -6.839 | 4.333 | 0.139 |
| D^RG^ | 12.302 | 7.214 | 0.112 |
| C | 355.474 | 83.377 | 0.001 |
| R^2^=0.60 Adj. R^2^=0.35 F-Stat.=2.44(0.07) | | | |
| RESET=0.25(0.63) B.P.G=7.89(0.44) | | | |
| J.B=0.33(0.85) D.W Stat. =2.1 | | | |
